# Culture and body image: subcultural variations in coping strategies and their associations with psychological distress among European Canadians and East Asian Canadians

**DOI:** 10.3389/fpsyg.2025.1596710

**Published:** 2025-08-13

**Authors:** Leah Rose Wojcik, Takahiko Masuda

**Affiliations:** Department of Psychology, University of Alberta, Edmonton, AB, Canada

**Keywords:** cultural differences, body image threats, appearance fixing, Positive Rational Acceptance, European Canadians, East Asian Canadians, psychological distress, culture and body image

## Abstract

Whether through friends, family, or social media, it is common to encounter situations that threaten body image. Threats to body image can lead individuals to question or perceive their appearance negatively if not coped with effectively. In turn, a negative body image is often associated with disordered eating patterns. Given the prevalence and severity of eating disorders globally, it is essential to gain insight into body image coping strategies to recognize adaptive ones while identifying those that may exacerbate body image concerns. With the significant cultural and ethnic diversity present in Canada, we investigated how two major ethnic groups cope with threats to their body image. We highlighted the importance of collecting data from underrepresented populations in psychological research and investigating nuanced variations across subgroups in multicultural societies. Participants included European Canadians (n = 156) and East Asian Canadians (*n* = 157) who rated the extent to which they used certain coping strategies in response to a body image threat. Cultural differences were examined in the usage of three coping strategies: appearance Fixing, Avoidance, and Positive Rational Acceptance. Linear regression analyses were conducted to explore relationships between the coping strategies and symptoms of stress, depression, and social anxiety. Results revealed a main effect of Culture on Appearance Fixing, with European Canadians more likely to endorse this strategy than East Asian Canadians when facing threats to body image. Conversely, East Asian Canadians tended to use Positive Rational Acceptance more than their European Canadian counterparts. In addition, we reported that overall, Appearance Fixing and Avoidance positively predicted symptoms of stress, depression, and social anxiety, and Positive Rational Acceptance emerged as a healthier way to cope, supporting the findings of similar previous works. The implications of culturally grounded research on body image and wellbeing are further discussed.

## 1 Introduction

It is crucial to develop coping mechanisms in order to effectively navigate the negative effects of stress. Coping mechanisms are defined as the thoughts and behaviors one uses to manage the demands of situations that are perceived as being stressful (Folkman and Moskowitz, 2004). Practicing adaptive stress coping strategies is beneficial to prevent stressors from interfering with daily functioning. Depending on the situation, (e.g., conflict with a co-worker, or a student who is about to write an exam) there are different types of stress coping mechanisms individuals may endorse. In addition to general stressful situations, it is common to experience stress resulting from situations threatening one's body image. Trying on a pair of pants that are now too small or being in the company of friends who have societally “ideal” body types are both examples of threats to body image.

Body image is one's personal view of their own body including their thoughts, perceptions, and beliefs (Cash, 2004). Furthermore, a threat to body image is defined as any situation that affects one's contentment with their body, leading to feelings of dissatisfaction with their appearance (Cash, 2004). One's body image is an essential factor to consider in the context of physical, psychological, and interpersonal functioning. It has been shown to be associated with less general secure attachment, more relationship intimacy anxiety, lower levels of overall confidence, higher social anxiety, and body dissatisfaction (Cash et al., 2005; Dames et al., 2024; Summers and Cougle, 2018). Body dissatisfaction has been shown to affect physical health, by increasing the risk of developing disturbed eating patterns such as binging and restricting, and potentially eating disorders (Andrés and Saldaña, 2014; Blodgett Salafia et al., 2015). Given the negative psychological and physical health outcomes associated with having a negative body image, it may be worth considering a proactive approach by identifying and implementing effective strategies to cope with body image threats in an adaptive way (Cash et al., 2005).

The Body Image Coping Strategies Inventory (BICSI) is a scale developed to measure the usage of coping strategies individuals would endorse in a situation posing a threat to their body image (Cash et al., 2005). The questionnaire consists of three subscales: appearance fixing, avoidance, and positive rational acceptance. Appearance Fixing involves coping with body image threats by altering one's appearance in some way (e.g., hiding perceived defects) (Cash et al., 2005). Avoidance involves withdrawing and not confronting the stressor (e.g., avoiding mirrors). Positive Rational Acceptance is a coping strategy involving adjusting one's mindset and reframing the situation in a more positive way (e.g., telling oneself there are more important things in life than looks). This scale has been included in various clinical and nonclinical studies, especially for body image and eating disorder research to investigate the relationships between coping strategies and disturbed eating behaviors, eating disorders, body shame, cosmetic surgery, and wellbeing (Callaghan et al., 2011; Choma et al., 2009; Dhurup and Nolan, 2014; Hrabosky et al., 2009).

In addition to the general patterns of responses found in the study by Cash et al., cultural differences in coping strategy usage between White and African American women were discussed, however the cultural differences were not a major focus of their study. The study referred to having a relatively small percentage of Asian participants (5.6%), so any unique patterns have not been reported. We maintain that exploring and deepening understanding of body image and coping mechanisms through a cultural lens is crucial because the approach to dealing with psychological and physical health risks could be divergent, even within a society, especially when that society is multicultural (Abdoli et al., 2024; Cheng, 1997). The current study aimed to address this gap in body image coping research by connecting to the existing work within cultural psychology. This goal resonates with the current debate in psychology regarding the extent of generalizability for existing psychological findings. Psychological research has most commonly been conducted in WEIRD (Western, Educated, Industrialized, Rich, Democratic) societies (Henrich et al., 2010). Researchers pointed out that relying on mainly WEIRD populations may limit the generalizability of research findings to other populations across the world. Due to the diverse cultural values, norms, and practices existing outside WEIRD cultures, theoretical frameworks within psychology and even clinical interventions may not be as applicable. This niche subpopulation of individuals cannot be used to generalize results to the entire rich, culturally diverse global population.

Conducting cultural research within a nation, especially one as multicultural and diverse as Canada, is also essential. To homogenize and classify individuals of all cultures under the single term based on nationality would fail to account for the hundreds of cultural and ethnic origins currently existing within Canada. Conducting more research into body image involving underrepresented populations, and investigating cultural variations is essential. At the national level, immigrants make up a significant part of the current Canadian population, as reflected in the 2021 Census. It revealed that about 1 in 4 individuals reported being a landed immigrant or permanent resident in Canada (Immigration Refugees and Citizenship Canada, 2022). Therefore, a large population of individuals living in Canada were born and often raised in a vastly different country with their own set of unique cultural and social norms, traditions/practices, languages, and even differing basic psychological processes. Immigrants in Canada are typically encouraged to maintain and express their culture, therefore, there would inevitably be pronounced cultural variations existing even within the nation. However, we are unaware of any published research that has addressed this specific issue in the context of body image research. To initiate body image coping research within the multicultural context in Canada, we selected East Asian Canadians as a target group by which we examined subcultural patterns of body image with their European Canadian counterparts. East Asians are a significant visible ethnic minority in Canada (accounting for over 6.3 percent of the total population), and therefore were selected as a target population for this research (Statistics Canada, 2024).

Cultural studies investigating the endorsement of various coping strategies have repeatedly found that Western cultures tend to endorse primary control coping, and Eastern cultures tend to endorse secondary control coping strategies to deal with stressful situations (Essau, 1992; Han et al., 2022; McCarty et al., 1999; Weisz et al., 1984). A primary control coping mechanism involves coping with a situation through direct influence on the external reality (Rothbaum et al., 1982). For example, choosing to actively make changes to one's home before having guests over in an attempt to improve it would be a primary control approach. Accepting the older, yet unique stylistic features of the home, viewing them from a positive point of view would be an example of secondary control coping. In this case, there was no overt action made to cope with the situation, and the individual adjusted their mindset instead.

Previous research has suggested that cultural variations in the ways individuals cope with stress stem from the socially held worldviews between cultures (Chun et al., 2006; Cross, 1995). Eastern cultures highly value social harmony, and individuals view themselves as interconnected through relationships, which is known as an interdependent social orientation (Kitayama et al., 2022; Markus and Kitayama, 2010; Masuda et al., 2008; Masuda, 2017; Varnum et al., 2010). Contrastingly, Western cultures (North Americans, such as European Canadians) have an independent social orientation, which manifests as viewing the self as separate from others and valuing autonomy and self-direction. Therefore, Eastern cultures typically endorse secondary control coping mechanisms, in order to preserve their social relationships and maintain harmony within society (Weisz et al., 1984). This allows for a less confrontational or less assertive way of managing conflict. On the other hand, Westerners place less emphasis on social harmony and more on the self, preferring primary control strategies (Weisz et al., 1984). Because Western cultures tend to cope with general stress through more direct, hands-on strategies (as seen in primary control), they may choose to endorse strategies that involve physically taking action and making changes to their appearance. Under this logic, because Eastern cultures (East Asians, such as Chinese, Korean, Japanese) tend to cope with stress without taking overt action and rather adjusting themselves to their environment (secondary control), this may have association with choosing strategies involving reframing their mindset and making an effort to think rationally, as seen in coping using Positive Rational Acceptance strategies.

As no published research to our knowledge has explored cultural differences specifically contrasting participants of European background with those of East Asian background when coping with threats to body image, our aim was to investigate the strategies that individuals typically endorse when experiencing a body image threat. Relationships between the three body image coping strategies and symptoms of social anxiety, depression, and stress were individually explored. Given that within Canada, individuals are encouraged to maintain and express their unique cultural values, we maintain that cultural differences would still be prominent within a single society. Although this research into cultural differences is the first attempt to delve into potential within cultural variation within Canada, we hypothesized that due to the cultural variations in self construal and associations with primary and secondary control, that there are substantial differences in the ways individuals cope with the stress of body image threats. More specifically, by using the Body Image Coping Strategies Inventory by Cash et al. (2004) we hypothesized that European Canadians would endorse Appearance Fixing to cope with threats to body image more often than their East Asian Canadian counterparts, to examine whether primary control and an independent self-construal are indeed associated with using a more direct coping strategy involving changing the situation. In contrast, we hypothesized that East Asian Canadians would endorse Positive Rational Acceptance to cope with these threats significantly more than European Canadians, because they hold an interdependent self-construal and tend to adjust themselves to a stressful situation rather than changing it. Additionally, we aimed to support previous works by hypothesizing that there would be relationships between the three coping styles and symptoms of stress, depression, and social anxiety. We investigated this by conducting three multivariate multiple linear regressions.

## 2 Materials and methods

### 2.1 Participants

The sample of participants for analysis consisted of 155 European Canadian (82 women, 73 men; *M*age = 19.9 years, SD = 3.01 years; age range = 18–37 years) and 157 East Asian Canadian (88 women, 69 men; *M*age = 19.8 years, SD = 1.88 years; age range = 18–28 years) undergraduate students. East Asian Canadians consisted of students with East Asian cultural backgrounds, including Chinese, Japanese, and Korean. European Canadian participants consisted of students with Northern European (Swedish, Danish) Southern European (Italian, Croatian, Serbian), Eastern European (Polish, Slovakian, Ukrainian, Hungarian, Romanian, Russian), and Western European (British Isles, French, German) origins. For the purposes of this study, a binary sex categorization (male/female) designated at birth was used for analyses. Participants were asked to indicate their sex, defined as a set of biological attributes that are associated with physical and physiological features. Although participants were also asked to identify their gender, three participants who self-identified as other than male or female had their data excluded from the study. This was due to their limited number, which would make grouping into a distinct category unfeasible for analysis. Additionally, 12 biracial participants identifying as both East Asian Canadian and European Canadian were excluded from the analysis, as the purpose of this study was to identify subcultural differences solely between the two distinct groups. Data from eight participants who identified as North American Indigenous were excluded from the analysis due to the small sample size. Given that Indigenous peoples in Canada have a distinct cultural background separate from European Canadians, their data were not included in the present study. One female participant was excluded from the analyses as an age-related outlier, belonging to a different generational cohort. One additional participant was excluded from the analysis due to completing less than 5% of the survey questions, resulting in insufficient data. In total, data from 25 participants were excluded from analysis. A priori power analysis was conducted using G^*^Power 3.1.9.7 (Faul et al., 2009) to test the main effects and interactions between Culture and Sex using medium effect size (*f* = 0.25), and an alpha level of.05. Results indicated that we needed a minimum of 128 participants in total to ensure a power of 0.80. The total number of participants for this study exceeded the criteria.

### 2.2 Measures

#### 2.2.1 Body image coping strategies

To test for cultural differences in the usage of coping strategies, The Body Image Coping Strategies Inventory (BICSI) was administered. The BICSI consists of 29 items to measure ways of coping in response to body image threats (Cash et al., 2005). The BICSI contains three subscales: appearance fixing, positive rational acceptance, and avoidance (Cash et al., 2005). Appearance Fixing involves altering one's appearance in some way, with a sample item being, “I do something to try to look more attractive.” Avoidance involves withdrawing and not confronting the stressor (e.g., “I withdraw and interact less with others”). Lastly, Positive Rational Acceptance involves reframing or adjusting one's mindset to deal with the stressor, as characterized by items such as, “I try to figure out why I am challenged or threatened by the situation.” Participants rated their usage of various coping strategies, using a 4-point Likert-type scale (0 = definitely not like me, 3=definitely like me), with higher mean subscale scores representing more frequent usage of a particular coping strategy. The subscale scores range from 0 to 4 (European Canadians α = 0.77, East Asian Canadians α = 0.74).

#### 2.2.2 Level of depressive symptoms

Participants completed the 20-item, Center for Epidemiologic Studies Depression Scale (CES-D) to measure levels of distress, as indicated by distress symptoms experienced in the past week (Radloff, 1977). The CES-D has been used in both clinical and non-clinical settings as a measure of overall wellbeing. Participants rated statements using a 4-point Likert-type scale (0 = rarely or none of the time, 3 = most or all of the time). Sample items include, “I felt lonely” and “I felt that people disliked me.” The scores can range from 0 to 60, with a total sum score of 16 points or more being considered depressed (European Canadians α = 0.91, East Asian Canadians α = 0.90).

#### 2.2.3 Stress level

The Perceived Stress Scale (PSS), consisting of 14-items, was rated by participants using a 5-point Likert-type scale (0 = never, 4 = very often). The scale was used to measure the degree to which situations in the past month are perceived as stressful (Cohen et al., 1983). Sample items include, “In the last month, how often have you found that you could not cope with all the things that you had to do?” and “In the last month, how often have you been upset because of something that happened unexpectedly?” Scores can range from 0 to 40, with greater scores being considered as having higher perceived stress levels (European Canadians α = 0.86, East Asian Canadians α = 0.81).

#### 2.2.4 Fear of negative evaluation

The Fear of Negative Evaluation Scale (FNE) is a 30-item measure used to assess participants' apprehension about what others think of them (Watson and Friend, 1969). Participants indicated whether each statement was true or false for themselves. Sample items include, “I become tense and jittery if I know I am being judged by my superiors.” and “I am afraid that others will not approve of me.” Scores range from 0 to 30, with greater scores representing a greater degree of fear of being negatively evaluated or judged by others (European Canadians KR-20 = 0.93, East Asian Canadians KR-20 = 0.89).

#### 2.2.5 Social avoidance and distress

The Social Avoidance and Distress Scale (SADS) consists of 28-items and was used to measure participants' levels of avoidance in social situations and distress (Watson and Friend, 1969). Participants indicated whether each statement was true or false for them personally. Sample items include, “I usually feel uncomfortable when I am in a group of people I don't know” and “I try to avoid formal social occasions.” Scores range from 0 to 28, with higher overall scores representing a greater tendency to experience distress resulting from social situations, and a tendency to avoid them (European Canadians KR-20 = 0.92, East Asian Canadians KR-20 = 0.91).

#### 2.2.6 Social interaction anxiety

The Social Interaction Anxiety Scale (SIAS) is a 20-item measure used to assess levels of anxiety related to interactions with other people (Mattick and Clarke, 1998). Participants rated statements on a 5-point Likert-type scale (0 = not at all characteristic or true of me, 4 = extremely characteristic or true of me). Sample items include, “I have difficulty making eye contact with others” and “I feel tense if I am alone with just one other person” Scores range from 0 to 80, with higher scores representing higher levels of social anxiety (European Canadians α = 0.92, East Asian Canadians α = 0.88).

#### 2.2.7 Social phobia

The Social Phobia Scale (SPS) consists of 30 items used to assess levels of anxiety while being observed, or anxiety experienced when engaging in activities in the presence of others (Mattick and Clarke, 1998). Participants rated statements on a 5-point Likert-type scale (0 = not at all characteristic or true of me, 4 = extremely characteristic or true of me). Sample items include, “I worry about shaking or trembling when I'm watched by other people” and “I would get tense if I had to carry a tray across a crowded cafeteria.” Scores range from 0 to 80, with higher scores representing higher levels of anxiety about being observed or scrutinized (European Canadians α = 0.95, East Asian Canadians α = 0.93).

### 2.3 Procedure

Participants registered for the current study online, through the research participation portal at the University of Alberta. The online video conferencing platform, Zoom was used for this study (Zoom Video Communications, n.d.). Upon registering, participants were sent a Zoom link to join at the corresponding time they signed up for the study session. Participants joined a private Zoom session online with one or two research assistants, who verified attendance and provided instructions throughout the session. Participants were informed that there were no direct benefits to participating, aside from receiving course credit for research participation. Participation in this study was completely voluntary, and participants were informed that they may withdraw at any time, for any reason. The survey was created using Qualtrics Software (Qualtrics, 2022). After a researcher explained the instructions, participants were sent a link to the Qualtrics questionnaire, which contained a consent form on the first page. Written informed consent was obtained from all participants. At the end of the study, participants were asked for their consent to use their data, which is mandatory in case a participant completes the study but chooses to have their data withdrawn. Data included in the dataset for this study were solely from participants who provided consent for data usage. If participants chose to not participate in the study, they were given the option of completing an alternative task in order to still receive course credit. The alternative task consisted of reading a research article and answering questions pertaining to the research methods used. All participants consented to participating in the study, and no participants chose to complete the alternative task.

After signing the consent form, participants proceeded to the survey, consisting of multiple questionnaires. The full questionnaire took no longer than 90 min to complete, and participants worked at their own pace with a researcher present on the Zoom call. Participants completed the BICSI, along with scales assessing for symptoms of social anxiety (SADS, SPS, SIAS, FNE), depression (CES-D), and stress (PSS). Prior to answering the BICSI, participants read a definition of “body image” and “body image threats” that was included in the original scale administration instructions. Under the definitions, participants read an additional paragraph asking them to think about how much each item is characteristic of how they usually cope or would probably cope with an event or situation that poses a threat or challenge to their body image feelings. Lastly, participants completed questions asking about demographic information including cultural and ethnic background, sex, gender, age, and number of years living in Canada. All participants were debriefed before exiting the Zoom session and consented to include their data in the study.

## 3 Results

### 3.1 Usage of body image coping strategies across cultural groups

Two-way ANOVAs were conducted to examine the main effects of culture and sex, as well as their interaction, on the usage of each coping strategy (Appearance Fixing, Avoidance, and Positive Rational Acceptance). We ran separate analyses for each individual coping strategy to examine the usage across cultural groups and sex.

**Figure 1:**
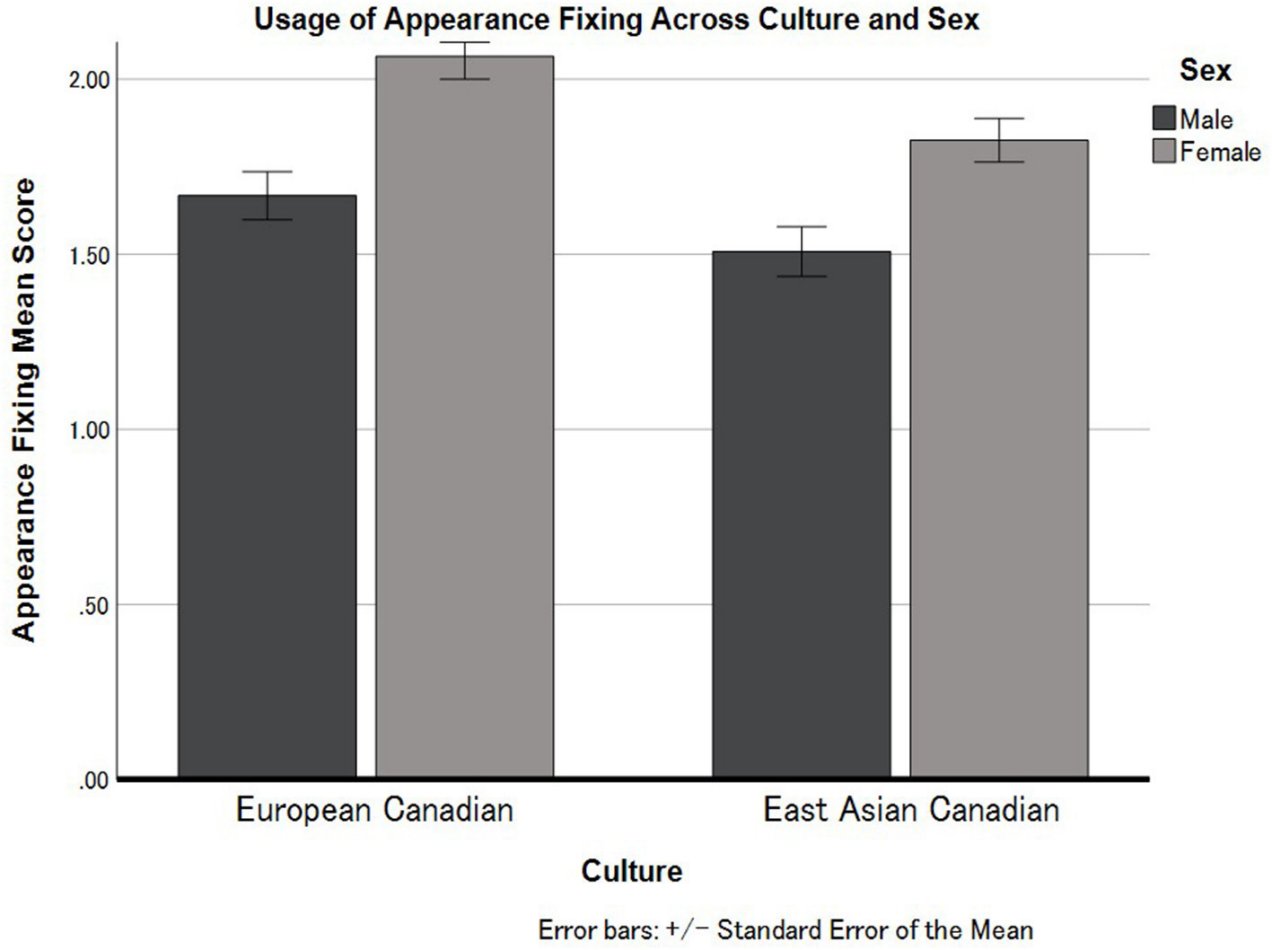
Cultural differences in the usage of Appearance Fixing in European Canadian and East Asian Canadian females and males. Error bars represent standard errors.

First, we conducted a 2 (Culture: European Canadian vs. East Asian Canadian) × 2 (Sex: Male vs. Female) ANOVA applying to Appearance Fixing. The results indicated that there was a main effect of Culture (Figure 1), indicating greater use of this strategy for European Canadians (*M* = 1.88, *SD* = 0.66) than East Asian Canadians (*M* = 1.69, *SD* = 0.56), *F*_(1, 312)_ = 8.90, *p* = 0.003, $\eta_{\text{p}}^{2}$ = 0.028. We also found a main effect of Sex, indicating greater usage of this strategy amongst females (*M* = 1.94, *SD* = 0.58) than males (*M* = 1.59, *SD* = 0.62) *F*_(1, 312)_ = 28.55, *p* < 0.001, $\eta_{\text{p}}^{2}$ = 0.085. The interaction effect between Culture and Sex for Appearance Fixing was not statistically significant, *F*_(1, 312)_ = 0.36, *p* < 0.356, $\eta_{\text{p}}^{2}$ = 0.001.

**Figure 2:**
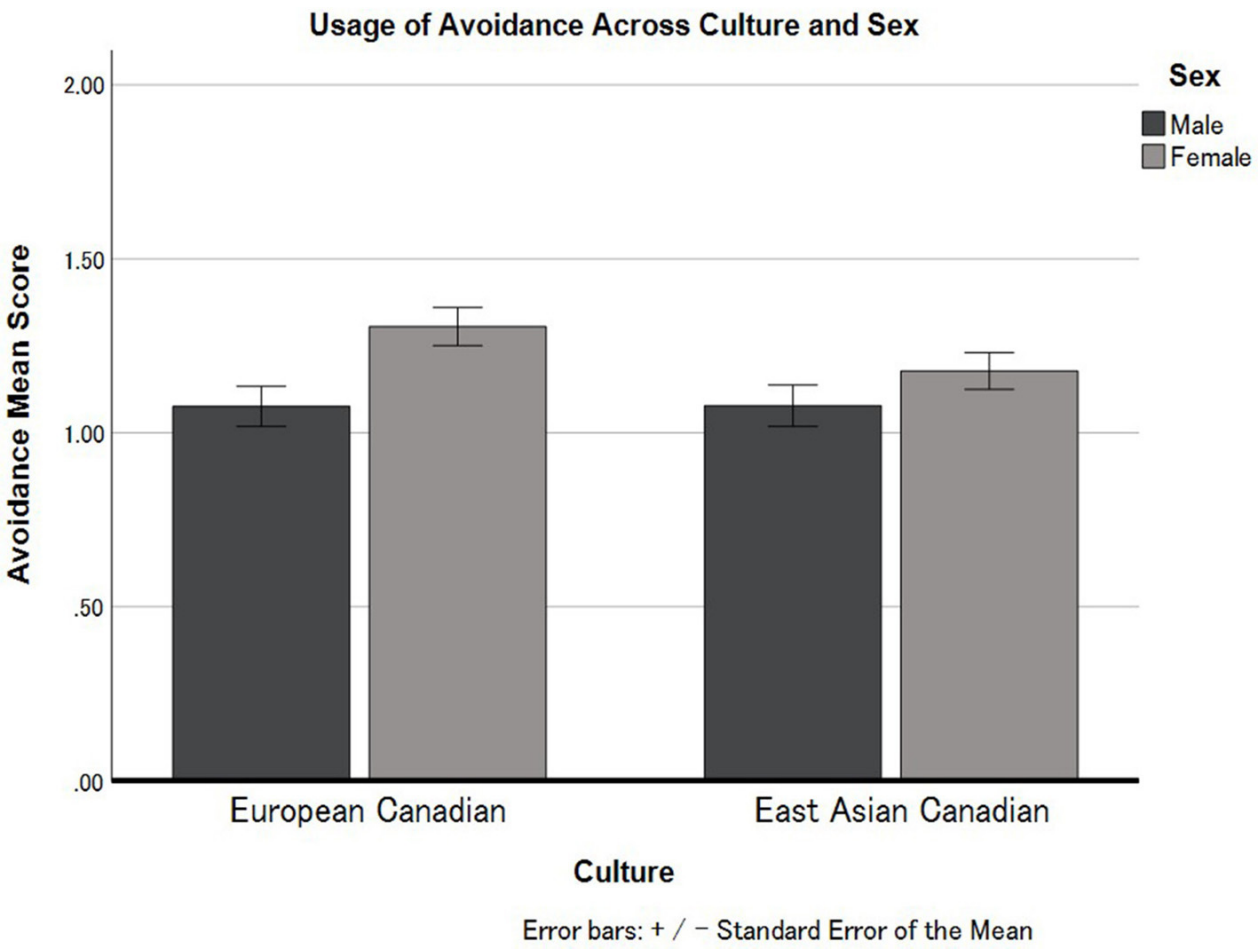
Cultural differences in the usage of Avoidance in European Canadian and East Asian Canadian females and males. Error bars represent standard errors.

Next, we conducted 2 (Culture: European Canadian vs. East Asian Canadian) × 2 (Sex: Male vs. Female) ANOVA applying to Avoidance (Figure 2). The results indicated that there is a main effect of Sex, indicating that females (*M* = 1.24, *SD* = 0.50) showed higher levels in the usage of Avoidance than males (*M* = 1.08, *SD* = 0.50), *F*_(1, 312)_ = 8.51, *p* < 0.004, $\eta_{\text{p}}^{2}$ = 0.027. No significant cultural differences were found in the usage of Avoidance coping strategies between European Canadians (*M* = 1.22, *SD* =.52), and East Asian Canadians (*M* = 1.13, *SD* = 0.45), *F*_(1, 312)_ = 1.24, *p* = 0.267, $\eta_{\text{p}}^{2}$ = 0.004. The interaction effect between Culture and Sex for Avoidance was not statistically significant, *F*_(1, 312)_ = 0.11, *p* = 0.743, $\eta_{\text{p}}^{2}$ < 0.001.

**Figure 3:**
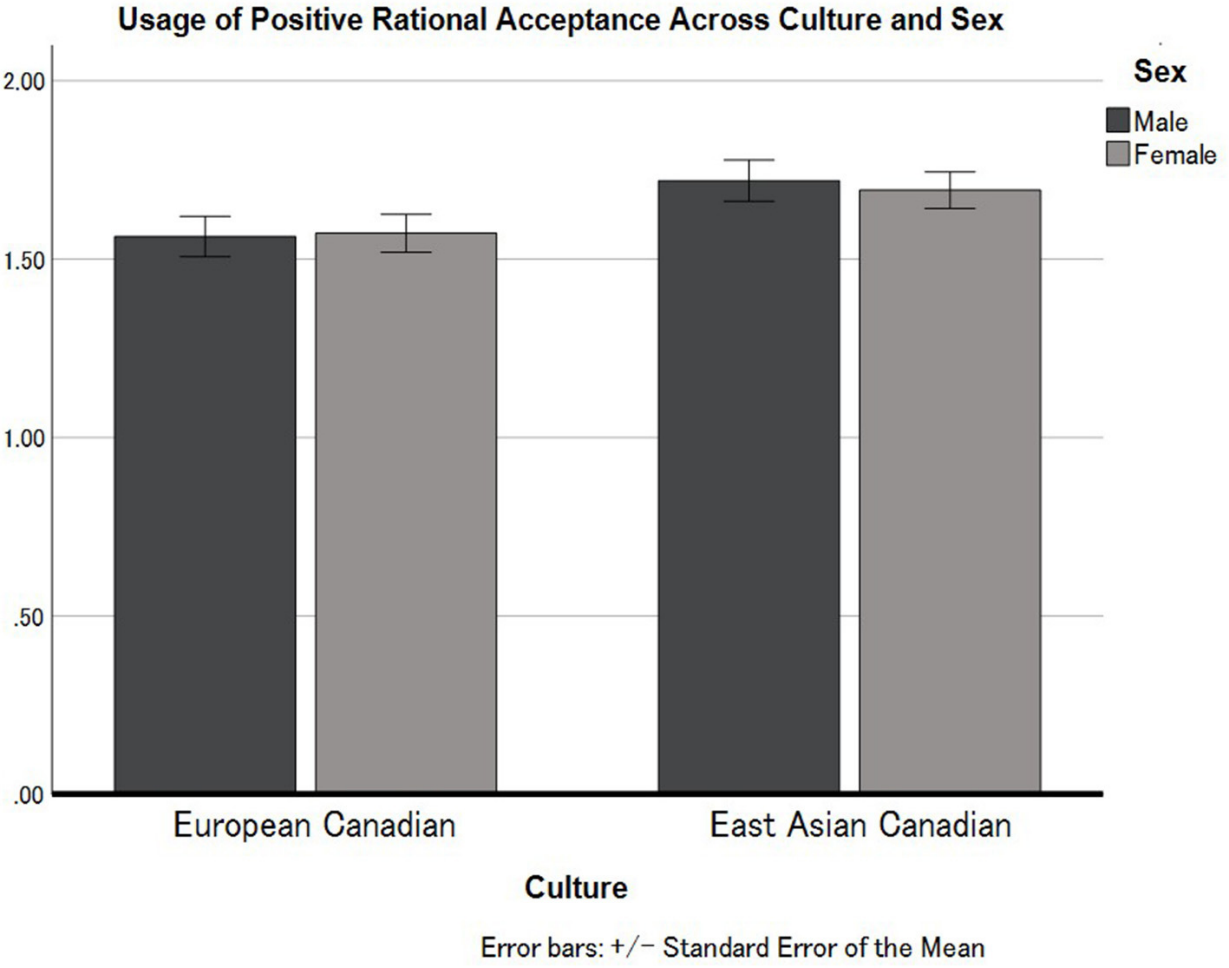
Cultural differences in the usage of Positive Rational Acceptance in European Canadian and East Asian Canadian females and males. Error bars represent standard errors.

Finally, we conducted another 2 (Culture: European Canadian vs. East Asian Canadian) × 2 (Sex: Male vs. Female) ANOVA applying to Positive Rational Acceptance. The results indicated that there is a main effect of Culture (Figure 3). East Asian Canadians (*M* = 1.70, *SD* = 0.42) overall showed higher levels in the usage of Positive Rational Acceptance compared to European Canadians (*M* = 1.57, *SD* = 0.53), *F*_(1, 312)_ = 6.40, *p* = 0.012, $\eta_{\text{p}}^{2}$ = 0.020. The main effect of Sex on Appearance Fixing was not statistically significant, *F*_(1, 312)_ = 0.03, *p* = 0.874, $\eta_{\text{p}}^{2}$ < 0.001. Additionally, the interaction effect between Culture and Sex for Positive Rational Acceptance was not statistically significant, *F*_(1, 312)_ = 0.11, *p* = 0.743, $\eta_{\text{p}}^{2}$ < 0.001.

### 3.2 Three multivariate multiple linear regression models

In the following subsections, we report results of three different Multilinear Multiple Linear Regression models to examine patterns identified in the respective ANOVA results. For your reference, we also reported a single comprehensive model among all the predictor variables including Appearance Fixing, Avoidance, and Positive Rational Acceptance. Please refer to Supplementary File 1 in details.

### 3.3 Multivariate multiple linear regression analysis between appearance fixing and symptoms of social anxiety, depression, and stress

A multivariate multiple linear regression was conducted to examine associations between three predictor variables (Culture, Sex, Appearance Fixing) and six dependent variables (Depression, Stress, Social Interaction Anxiety, Social Phobia, Fear of Negative Evaluation, Social Avoidance and Distress). Collinearity diagnosis indicated that the Variance Inflation Factor (VIF) of Culture and Sex to Appearance Fixing (VIF = 1.00, VIF = 1.00 respectively), Culture and Appearance Fixing to Sex (VIF = 1.03, VIF = 1.03 respectively), Sex and Appearance Fixing to Culture (VIF = 1.09, VIF = 1.09 respectively) were not indicative of multicollinearity. We chose to use Pillai's Trace as the multivariate test because of its robustness to violations of assumptions, including unequal covariance matrices and deviations from multivariate normality (Olson, 1976). Compared to other multivariate test statistics, Pillai's Trace is typically considered a more robust and conservative option, and therefore is recommended for general use.

Results indicated that the multivariate effect of Appearance Fixing was statistically significant, Pillai's Trace = 0.173, *F*_(6, 300)_ = 10.46, *p* < 0.001, $\eta_{\text{p}}^{2}$ = 0.173. The multivariate effect of Culture also showed a significant multivariate effect, Pillai's Trace = 0.116, *F*_(6, 300)_ = 6.54, *p* < 0.001, $\eta_{\text{p}}^{2}$ = 0.116. The multivariate effect of Sex was not statistically significant, Pillai's Trace =.036, *F*_(6, 300)_ = 1.84, *p* = 0.090, $\eta_{\text{p}}^{2}$ = 0.036. The interaction between Sex and Culture was statistically significant, Pillai's Trace = 0.046, *F*_(6, 300)_ = 2.38, *p* = 0.029, $\eta_{\text{p}}^{2}$ = 0.046, indicating that European Canadian females are more likely to report higher values for Social Interaction Anxiety (SIAS) compared to males. Such a pattern was not observed among East Asian Canadians (Table 1).

**Table 1:** Multivariate effects for appearance fixing.

**Effect**	**Pillai's trace**	***F***	**Hypothesis *df***	**Error *df***	***P***	**Partial η^2^**
Appearance fixing	0.173	10.459	6	300	< 0.001	0.173
Culture	0.116	6.542	6	300	< 0.001	0.116
Sex	0.036	1.844	6	300	0.090	0.036
Culture × sex	0.046	2.384	6	300	0.029	0.046

Results indicated that 11.6% of the variance in Perceived Stress (PSS) scores can be accounted for by the three variables (Culture, Sex, Appearance Fixing) collectively (Adjusted *R*^2^ = 0.116). Looking at the unique contributions of the predictors, the result shows that Positive Rational Acceptance predicted Perceived Stress (PSS) (β = 0.22*, t* = 4.36*, p* < 0.001). The other predictor variables did not significantly contribute toward explaining the association with PSS. Next, 9% of the variance in Social Phobia scores can be accounted for by the three variables collectively (Adjusted R^2^ = 0.090). Looking at the unique contributions of the predictors, the result shows that Positive Rational Acceptance predicted Social Phobia (SPS) (β = 0.32*, t* = 3.93*, p* < 0.001). The other predictor variables did not significantly contribute to explain the association with SPS. Third, 9.2% of the variance in Social Interaction Anxiety (SIAS) scores can be accounted for by the three variables (Culture, Sex, Appearance Fixing) collectively (Adjusted *R*^2^ = 0.092). Looking at the unique contributions of the predictors, the result shows that Appearance Fixing predicted Social Interaction Anxiety (SIAS) (β = 0.25*, t* = 3.79*, p* < 0.001). The other predictor variables did not significantly contribute to explain the association with SIAS. Fourth, 10.8% of the variance in Depression (CES-D) scores can be accounted for by the three variables (Culture, Sex, Appearance Fixing) collectively (Adjusted *R*^2^ = 0.108). Looking at the unique contributions of the predictors, the result shows that Appearance Fixing predicted Depression (CES-D) (β = 0.32*, t* = 4.44*, p* < 0.001). The other predictor variables did not significantly contribute to explain the association with CES-D. Fifth, 12.4% of the variance in Fear of Negative Evaluation (FNE) scores can be accounted for by the three variables (Culture, Sex, Appearance Fixing) collectively (Adjusted *R*^2^ = 0.124). Looking at the unique contributions of the predictors, the result shows that Appearance Fixing predicted Fear of Negative Evaluation (FNE) (β= −0.20*, t* = 4.58*, p* < 0.001). Additionally, Culture predicted FNE (β = 0.15*, t* = 2.20*, p* = 0.029), suggesting that East Asian Canadians are more likely to report higher levels of fear of negative evaluation compared to their European Canadian counterparts. Finally, 4.4% of the variance in Social Avoidance and Distress (SADS) scores can be accounted for by the three variables (Culture, Sex, Appearance Fixing) collectively (Adjusted *R*^2^ = 0.044). Looking at the unique contributions of the predictors, the result shows that Culture predicted SADS (β = −0.49*, t* = −3.00*, p* = 0.003), suggesting that East Asian Canadians reported higher scores of social avoidance and distress compared to European Canadians. Additionally, Sex predicted SADS (β = −0.355*, t* = −2.05*, p* = 0.042) suggesting that females reported higher scores of social avoidance and distress than males (Table 2).

**Table 2:** Parameter estimates.

**Dependent variable**	**Predictor**	**B**	**SE**	***t***	***p***	**Partial η^2^**
SADS	Appearance fixing	−0.016	0.103	0.151	0.880	< 0.001
	Culture	−0.492	0.164	2.997	0.003	0.029
	Sex	−0.355	0.173	2.046	0.042	0.014
	Culture × sex	0.158	0.242	0.655	0.513	0.001
FNE	Appearance fixing	−0.195	0.043	4.579	< 0.001	0.064
	Culture	0.148	0.068	2.194	0.029	0.016
	Sex	0.148	0.071	0.140	0.889	< 0.001
	Culture × sex	0.155	0.100	1.554	0.121	0.008
PSS	Appearance fixing	0.218	0.050	4.358	< 0.001	0.059
	Culture	0.128	0.080	1.606	0.109	0.008
	Sex	−0.097	0.084	1.159	0.247	0.004
	Culture × sex	−0.181	0.117	1.544	0.124	0.008
SPS	Appearance fixing	0.318	0.081	3.933	< 0.001	0.048
	Culture	−0.165	0.129	1.284	0.200	0.005
	Sex	−0.062	0.136	0.453	0.651	0.001
	Culture × Sex	−0.269	0.190	1.420	0.157	0.007
SIAS	Appearance fixing	0.253	0.067	3.788	< 0.001	0.045
	Culture	−0.115	0.106	1.080	0.281	0.004
	Sex	−0.037	0.112	0.329	0.742	−0.258
	Culture × Sex	−0.271	0.157	1.731	0.085	0.010
CESD	Appearance Fixing	0.316	0.071	4.435	< 0.001	0.061
	Culture	0.316	0.114	1.593	0.112	0.008
	Sex	0.181	0.120	1.301	0.194	0.006
	Culture × Sex	−0.134	0.167	0.802	0.423	0.002

SADS, social avoidance and distress scale; FNE, fear of negative evaluation; PSS, perceived stress scale; SPS, social phobia scale; SIAS, social interaction anxiety scale; CESD, center for epidemiologic studies depression scale.

Overall, this multivariate multiple regression model suggests that Appearance Fixing positively predicts symptoms of social anxiety, depression, and stress. The contributions of the other predictor variables were less pronounced after accounting for shared variance among all predictors.

### 3.4 Multivariate multiple linear regression analysis between avoidance and symptoms of social anxiety, depression, and stress

A multivariate multiple linear regression was conducted to examine associations between three predictor variables (Culture, Sex, Avoidance) and six dependent variables (Depression, Stress, Social Interaction Anxiety, Social Phobia, Fear of Negative Evaluation, Social Avoidance and Distress). Collinearity diagnosis indicated that the Variance Inflation Factor (VIF) of Culture and Sex to Avoidance (VIF = 1.00, VIF = 1.00 respectively), Culture and Avoidance to Sex (VIF = 1.00, VIF = 1.0 respectively), Sex and Avoidance to Culture (VIF = 1.03, VIF = 1.03 respectively) were not indicative of multicollinearity.

The results indicated that the multivariate effect of Avoidance showed a significant multivariate effect, Pillai's Trace = 0.234, *F*_(6, 300)_ = 15.24, *p* < 0.001, $\eta_{\text{p}}^{2}$ = 0.234. The multivariate effect of Culture also had a statistically significant multivariate effect on the combined dependent variables, Pillai's Trace = 0.126, *F*_(6, 300)_ = 7.23, *p* < 0.001, $\eta_{\text{p}}^{2}$ = 0.126. The multivariate effect of Sex was statistically significant, Pillai's Trace = 0.052, *F*_(6, 300)_ = 2.73, *p* < 0.013, $\eta_{\text{p}}^{2}$ = 0.052. An interaction between Sex and Culture was found, Pillai's Trace = 0.041, *F*_(6, 300)_ = 2.16, *p* < 0.047, $\eta_{\text{p}}^{2}$ = 0.041. Again, indicating that European Canadian females are more likely to report higher values for Social Interaction Anxiety (SIAS) compared to males. Such a pattern was not observed among East Asian Canadians (Table 3).

**Table 3:** Multivariate effects for avoidance.

**Effect**	**Pillai's trace**	***F***	**Hypothesis *df***	**Error *df***	***P***	**Partial η^2^**
Avoidance	0.234	15.237	6	300	< 0.001	0.234
Culture	0.126	7.225	6	300	< 0.001	0.126
Sex	0.052	2.730	6	300	0.013	0.052
Culture × sex	0.041	2.159	6	300	0.047	0.041

Results indicated that 20.4% of the variance in Perceived Stress (PSS) scores can be accounted for by the three variables (Culture, Sex, Avoidance) collectively (Adjusted *R*^2^ = 0.204). Looking at the unique contributions of the predictors, the result shows that Avoidance predicted Perceived Stress (PSS) (β = 0.042*, t* = 7.41, *p* < 0.001). The other predictor variables did not significantly contribute to explain the association with PSS. Next, results indicated that 15.5% of the variance in Social Phobia (SPS) scores can be accounted for by the three variables collectively (Adjusted *R*^2^ = 0.155). Looking at the unique contributions of the predictors, the result shows that Avoidance predicted Social Phobia (SPS) (β = 0.58*, t* = 6.32*, p* < 0.001). The other predictor variables did not significantly contribute to explain the association with SPS. Third, 18.1% of the variance in Social Interaction Anxiety (SIAS) scores can be accounted for by the three variables collectively (Adjusted *R*^2^ = 0.181). Looking at the unique contributions of the predictors, the result shows that Avoidance predicted Social Interaction Anxiety (SIAS) (β = 0.53*, t* = 7.01*, p* < 0.001). The other predictor variables did not significantly contribute to explain the association with SIAS. Fourth, 20% of the variance in Depression (CES-D) scores can be accounted for by the three variables (Culture, Sex, Avoidance) collectively (Adjusted *R*^2^ = 0.200). Looking at the unique contributions of the predictors, the result shows that Avoidance predicted Depression (CES-D) (β = 0.60*, t* = 7.55*, p* < 0.001). The other predictor variables did not significantly contribute to explain the association with CES-D. Fifth, 14.2% of the variance in Fear of Negative Evaluation (FNE) scores can be accounted for by the three variables (Culture, Sex, Avoidance) collectively (Adjusted *R*^2^ = 0.142). Looking at the unique contributions of the predictors, the result shows that Avoidance predicted Fear of Negative Evaluation (FNE) (β = −0.26*, t* = 5.28*, p* < 0.001). Additionally, Culture predicted FNE (β = 0.14*, t* = 2.03*, p* = 0.043), suggesting that East Asian Canadians are more likely to report higher levels of fear of negative evaluation compared to their European Canadian counterparts. Finally, 14.9% of the variance in Social Avoidance and Distress (SADS) scores can be accounted for by the three variables (Culture, Sex, Avoidance) collectively (Adjusted *R*^2^ = 0.142). Looking at the unique contributions of the predictors, the result shows that Avoidance predicted SADS (β = 0.70*, t* = 6.14*, p* < 0.001) (Table 4).

**Table 4:** Parameter estimates.

**Dependent variable**	**Predictor**	**B**	**SE**	***t***	***P***	**Partial η^2^**
SADS	Avoidance	0.706	0.115	6.139	< 0.001	0.110
	Culture	−0.279	0.161	1.735	0.084	0.045
	Sex	−0.279	0.161	1.735	0.084	0.010
	Culture x Sex	0.246	0.228	1.076	0.283	0.004
FNE	Avoidance	−0.262	0.050	5.276	< 0.001	0.084
	Culture	0.135	0.066	2.034	0.043	0.013
	Sex	0.046	0.069	0.658	0.511	0.001
	Culture × Sex	0.139	0.099	1.412	0.159	0.006
PSS	Avoidance	0.416	0.056	7.411	< 0.001	0.153
	Culture	0.127	0.075	1.694	0.091	0.009
	Sex	−0.125	0.079	1.597	0.111	0.008
	Culture × Sex	−0.149	0.111	1.336	0.182	0.006
SPS	Avoidance	0.582	0.092	6.321	< 0.001	0.116
	Culture	−0.163	0.123	1.326	0.186	0.006
	Sex	−0.105	0.129	0.812	0.417	0.002
	Culture × sex	−0.225	0.183	1.231	0.219	0.005
SIAS	Avoidance	0.525	0.075	7.006	< 0.001	0.139
	Culture	−0.121	0.100	1.208	0.228	0.005
	Sex	−0.065	0.105	0.621	0.535	0.001
	Culture × Sex	−0.228	0.149	1.534	0.126	0.008
CESD	Avoidance	0.602	0.080	7.554	< 0.001	0.158
	Culture	0.180	0.107	1.684	0.093	0.009
	Sex	−0.196	0.112	1.758	0.080	0.010
	Culture × Sex	−0.087	0.158	0.551	0.582	0.001

SADS, social avoidance and distress scale; FNE, fear of negative evaluation; PSS, perceived stress scale; SPS, social phobia scale; SIAS, social interaction anxiety scale; CESD, center for epidemiologic studies depression scale.

Overall, similar to Appearance Fixing, this multivariate multiple regression model suggests that Avoidance positively predicts symptoms of social anxiety, depression, and stress. The contributions of the other predictor variables were less pronounced after accounting for shared variance among all predictors.

### 3.5 Multivariate multiple linear regression analyses between Positive Rational Acceptance and symptoms of social anxiety, depression, and stress

A multivariate multiple linear regression was conducted to examine associations between three predictor variables (Culture, Sex, Positive Rational Acceptance) and six dependent variables (Depression, Stress, Social Interaction Anxiety, Social Phobia, Fear of Negative Evaluation, Social Avoidance and Distress). Collinearity diagnosis indicated that the Variance Inflation Factor (VIF) of Culture and Sex to Positive Rational Acceptance (VIF = 1.00, VIF = 1.00 respectively), Culture and Positive Rational Acceptance to Sex (VIF = 1.02, VIF = 1.02 respectively), Sex and Positive Rational Acceptance to Culture (VIF = 1.00, VIF = 1.00 respectively) were not indicative of multicollinearity.

Results indicated that the multivariate effect of Positive Rational Acceptance was statistically significant, Pillai's Trace = 0.097, *F*_(6, 300)_ = 5.35, *p* < 0.001, $\eta_{\text{p}}^{2}$ = 0.097. The multivariate effect of Culture also had a significant multivariate effect on the combined dependent variables, Pillai's Trace = 0.115, *F*_(6, 300)_ = 6.50, *p* < 0.001, $\eta_{\text{p}}^{2}$ = 0.115. The multivariate effect of Sex was also statistically significant, Pillai's Trace = 0.077, *F*_(6, 300)_ = 4.16, *p* < 0.001, $\eta_{\text{p}}^{2}$ = 0.077. An interaction effect between Sex and Culture was found, Pillai's Trace = 0.045, *F*_(6, 300)_ = 2.34, *p* < 0.032, $\eta_{\text{p}}^{2}$ = 0.045. This indicates that European Canadian females are more likely to report higher values for Social Interaction Anxiety (SIAS) compared to males. Such a pattern was not observed among East Asian Canadians (Table 5).

**Table 5:** Multivariate effects for Positive Rational Acceptance.

**Effect**	**Pillai's trace**	***F***	**Hypothesis *df***	**Error *df***	***P***	**Partial η^2^**
Positive Rational Acceptance	0.097	5.349	6	300	< 0.001	0.097
Culture	0.115	6.498	6	300	< 0.001	0.115
Sex	0.077	4.157	6	300	< 0.001	0.077
Culture × Sex	0.045	2.336	6	300	0.032	0.045

Results indicated that 10.2% of the variance in Perceived Stress (PSS) scores can be accounted for by the three variables (Culture, Sex, Positive Rational Acceptance) collectively (Adjusted *R*^2^ = 0.102). Looking at the unique contributions of the predictors, the result shows that Positive Rational Acceptance predicted Perceived Stress (PSS) (β = −0.23*, t* = 3.72, *p* < 0.001). The other predictor variables did not significantly contribute to explain the association with PSS. Second, 5.3% of the variance in Social Phobia scores can be accounted for by the three variables collectively (Adjusted *R*^2^ = 0.053). However for Social Phobia, none of the predictors showed a statistically significant effect when included together, which may be due to overlapping variance among predictors rather than no existing relationship. Third, 8% of the variance in Social Interaction Anxiety (SIAS) scores can be accounted for by the three variables (Culture, Sex, Positive Rational Acceptance) collectively (Adjusted *R*^2^ = 0.080). Looking at the unique contributions of the predictors, Positive Rational Acceptance predicted Social Interaction Anxiety (SIAS) (β = −0.26*, t* = 3.22 *p* = 0.001). Fourth, 11.7% of the variance in Depression (CES-D) scores can be accounted for by the three variables (Culture, Sex, Positive Rational Acceptance) collectively (Adjusted *R*^2^ = 0.117). Looking at the unique contributions of the predictors, Positive Rational Acceptance predicted Depression (CES-D) (β = −0.41*, t* = 4.81*, p* < 0.001). Additionally, Sex predicted Depression (β = −0.25*, t* = 2.10*, p* = 0.037). Fifth, 6.3% of the variance in Fear of Negative Evaluation (FNE) scores can be accounted for by the three variables (Culture, Sex, Positive Rational Acceptance) collectively (Adjusted *R*^2^ = 0.063). However for Fear of Negative Evaluation, none of the predictors showed a statistically significant effect when included together, which may be due to overlapping variance among predictors rather than no existing relationship. Finally, 8.9% of the variance in Depression (CES-D) scores can be accounted for by the three variables (Culture, Sex, Positive Rational Acceptance) collectively (Adjusted *R*^2^ = 0.089). Looking at the unique contributions of the predictors, Positive Rational Acceptance predicted Social Avoidance and Distress (SADS) (β = −0.48*, t* = 3.89*, p* < 0.001). Additionally, Culture predicted Social Avoidance and Distress (β = −0.55*, t* = 3.48*, p* = 0.038). Sex also predicted Social Avoidance and Distress (β = −0.34*, t* = 2.13*, p* = 0.043). This suggests that females tend to report higher scores in social avoidance than males, and East Asian Canadians report higher scores than European Canadians (Table 6).

**Table 6:** Parameter estimates.

**Dependent variable**	**Predictor**	**B**	**SE**	***t***	***P***	**Partial η^2^**
SADS	Positive Rational Acceptance	−0.475	0.122	3.885	< 0.001	0.047
	Culture	−0.553	0.159	3.475	< 0.001	0.038
	Sex	−0.337	0.166	2.028	0.043	0.013
	Culture x Sex	0.143	0.236	0.606	0.545	0.001
FNE	Positive Rational Acceptance	0.004	0.053	0.071	0.943	< 0.001
	Culture	0.102	0.069	1.474	0.142	0.007
	Sex	0.072	0.072	0.991	0.323	0.003
	Culture x Sex	0.171	0.103	1.666	0.097	0.009
PSS	Positive Rational Acceptance	−0.228	0.061	3.716	< 0.001	0.043
	Culture	0.153	0.080	1.915	0.056	0.012
	Sex	−0.161	0.083	1.931	0.054	0.012
	Culture × Sex	−0.208	0.118	1.757	0.080	0.010
SPS	Positive Rational Acceptance	−0.175	0.100	1.750	0.081	0.010
	Culture	−0.110	0.130	0.847	0.398	0.002
	Sex	−0.158	0.136	1.161	0.247	0.004
	Culture × Sex	−0.302	0.193	1.564	0.119	0.008
SIAS	Positive Rational Acceptance	−0.262	0.082	3.215	0.001	0.033
	Culture	−0.086	0.106	0.809	0.419	0.002
	Sex	−0.110	0.111	0.996	0.320	0.003
	Culture × Sex	−0.302	0.157	1.916	0.056	0.012
CESD	Positive rational acceptance	−0.414	0.086	4.805	< 0.001	0.070
	Culture	0.207	0.112	1.843	0.066	0.011
	Sex	−0.245	0.117	2.097	0.037	0.014
	Culture × Sex	−0.175	0.166	1.056	0.292	0.004

SADS, social avoidance and distress scale; FNE, fear of negative evaluation; PSS, perceived stress scale; SPS, social phobia scale; SIAS, social interaction anxiety scale; CESD, center for epidemiologic studies depression scale.

Different from Appearance Fixing and Avoidance, this multivariate model found that Positive Rational Acceptance negatively predicts symptoms of social anxiety, depression and stress. This suggests that Positive Rational Acceptance may play an important role as an adaptive coping mechanism. The contributions of the other predictor variables were less pronounced after accounting for shared variance among all predictors.

## 4 Discussion

The current study investigated differences between European Canadians and East Asian Canadians, in coping with threats to body image by using Positive Rational Acceptance, Appearance Fixing, and Avoidance. Additionally, it explored relationships between these coping strategies and symptoms of depression, stress, and social anxiety.

As expected, European Canadians and East Asian Canadians differed in their usage of Appearance Fixing and Positive Rational Acceptance strategies. European Canadians scored significantly higher on Appearance Fixing than East Asian Canadians in response to body image threats, and East Asian Canadians endorsed Positive Rational Acceptance to a greater extent. This finding may be explained by previously established frameworks in stress coping across Eastern and Western cultures, which have consistently demonstrated that East Asians tend to generally prefer secondary coping in stressful scenarios (which involves acceptance and reframing the situation by adjusting one's mindset to the situation) rather than adjusting the situation to oneself, as seen in primary coping (Essau, 1992; Han et al., 2022; McCarty et al., 1999). This could explain the East Asian Canadian tendency to lean toward a similar type of secondary control approach as seen in Positive Rational Acceptance, which also involves situational reframing, and acceptance (Cash et al., 2005). The research on primary and secondary stress coping and self-construals may also help explain the tendency for European Canadians to endorse Appearance Fixing strategies more than Positive Rational Acceptance (Essau and Trommsdorff, 1996; Han et al., 2022; McCarty et al., 1999). Rather than adjusting one's mindset to the situation or stressor, Appearance Fixing involves actively altering one's appearance, to cope with the stress resulting from a threat to body image. In this sense, one is actively altering the situation (i.e., how they appear), rather than adjusting their mindset to deal with the situation (Cash et al., 2005). This coping approach seems to be associated with primary control, which is more common in Western societies holding an independent self-construal.

Sex differences were found in the usage of Appearance Fixing and Avoidance, in that women from both cultures tended to use this coping strategy more than males, which is consistent with the research by Cash et al. (2004). This may be explained by research demonstrating that women are often being socialized to prioritize their appearance and to seek validation based on their attractiveness (Neagu and Rainer, 2005). In contrast, men tend to be socialized to prioritize areas other than physical appearance, such as independence and toughness. Therefore, men may not feel the need to the same extent as women, to alter their physical appearance. Additionally, women may face greater societal pressure than men to conform to beauty standards, which can lead to body dissatisfaction and inevitably, the desire to alter their appearance (Helfert and Warschburger, 2013).

In contrast with Appearance Fixing and Avoidance, only Positive Rational Acceptance was found to predict lower levels of depressive symptoms, social interaction anxiety, and stress. These findings are supported by past research showing that strategies such as positive reframing and acceptance tend to help alleviate symptoms of stress and are positively correlated with psychological wellbeing (Guszkowska and Dabrowska-Zimakowska, 2022). Khoshkerdar and Raeisi (2020) investigated the effects of a mindfulness-based stress reduction intervention on adolescent girls with dysfunctional eating attitudes and found that it resulted in a reduction in body image concerns. This mindfulness intervention involved a focus on acceptance, elimination of dysfunctional negative thoughts, and avoiding self-blame. Positive Rational Acceptance similarly involves these aspects by emphasizing acceptance of the situation, reframing it, and engaging in rational self-talk about one's appearance (Cash et al., 2005). Therefore, adopting Positive Rational Acceptance as a coping strategy when faced with a threat to body image could be a way of directly reducing the negative impact of the threat and help protect overall wellbeing. Another study found that individuals who coped with body image challenges using Positive Rational Acceptance received higher scores on mindfulness subscales (Mackenzie, 2012). Individuals who tended to use more Appearance Fixing and Avoidance strategies scored lower on mindfulness subscales, which further highlights the presence and importance of mindfulness in Positive Rational Acceptance (Mackenzie, 2012). This research, in combination with the current study may suggest that Positive Rational Acceptance, which is closely related to mindfulness, may overall be a more adaptive and healthier way of coping.

Results of the multivariate multiple linear regressions conveyed that Appearance Fixing and Avoidance predicted higher levels of psychological distress symptoms related to social anxiety, depression, and stress. Specifically, they predicted levels of perceived stress, depression, fear of negative evaluation, social interaction, social phobia. These findings are also consistent with past research like the study by Summers and Cougle (2018) on Appearance-related safety-behaviors which involve checking, hiding, fixing, and reducing threats associated with their perceived flaws. Appearance-related safety behaviors were found to be positively associated with social anxiety and body dissatisfaction. This supports the current study's findings on Appearance Fixing and Avoidance coping strategies, which similarly to appearance-related safety-behaviors, are associated with symptoms of social anxiety. This further demonstrates the potentially damaging effects of Appearance Fixing and Avoidance coping strategies on psychological and physical health.

### 4.1 Implications

The current study addressed an area not yet studied in stress coping research and has several implications. Firstly, it offers new insight into cultural variations within a highly multicultural nation by expanding psychological research beyond WEIRD societies. Adding to the literature base contributes to the goal to increase generalizability of findings by collecting data from different cultures (Henrich et al., 2010). Reporting nuanced cultural variations has become increasingly common within psychological research. By investigating cultural differences in body image and coping, this work opens up a new line of interdisciplinary research by integrating both cultural and health/clinical psychology. The study aimed to encourage the cultural competence of practitioners in the mental health field. With increasing cultural research, practitioners are likely to become more culturally-informed and learn about the unique values and coping strategies employed across cultures. Given the unique demographic in Canada, working with diverse populations is an essential part of work for most practitioners. Having a deeper cultural understanding will ultimately foster more trusting relationships with patients, and result in higher quality, tailored treatments that meet their unique needs.

Second, we discussed many of the negative physical and psychological effects related to a negative body image and maladaptive coping, yet the most serious outcomes have not been addressed. We believe the study can have implications for future studies in body image and eating disorders. Over 1.7 million Canadians are diagnosed with an eating disorder, and since 1990, eating disorders have been on the rise in some East Asian countries (Galmiche et al., 2019; Li et al., 2021). Eating disorders have especially been increasing globally since the COVID-19 pandemic (van Hoeken and Hoek, 2020). In most extreme cases, eating disorders like anorexia nervosa and bulimia are life-threatening and eating disorders account for the loss of over 3.3 million lives per year (Rodgers et al., 2020; van Hoeken and Hoek, 2020). Individuals diagnosed with anorexia nervosa, bulimia nervosa, and body dysmorphic disorder have shown greater usage of Appearance Fixing compared to clinical controls (Hrabosky et al., 2009). Given the significant physical effects that may occur due to an accumulation of threats to body image, it is vital to put into practice beneficial coping strategies to protect individuals from these damaging effects.

In current psychotherapy treatment approaches, there is an increasing interest in the application of mindfulness, and therapies based on Eastern traditions for mental health issues, similar to the roots of Positive Rational Acceptance (Keng et al., 2011). Acceptance and Commitment Therapy (ACT), emphasizes healing through acceptance and mindfulness, which is a key element in Positive Rational Acceptance (Fung, 2015). Although research investigating the effect of ACT on treating body image disorders is limited, a systematic review found that this approach shows potential when it comes to reducing constant thoughts regarding eating, weight, and body appearance post-intervention (Griffiths et al., 2018). Although rudimentary, the current study hopes to inform the application of therapies like ACT, especially for eating and body image related disorders, and hopes to encourage strategies related to Positive Rational Acceptance as a coping mechanism in response to body image threats.

### 4.2 Study limitations

There are several limitations for the current study that future research could aim to resolve. First, the current study investigated two cultural groups within one country. Many East Asian Canadian participants moved to Canada at a young age, therefore were immersed in Western culture for much longer than those who only recently moved to Canada. This may limit the degree of the cultural findings, as the two groups may be more similar, compared to studying European background Canadians and individuals born and currently living in East Asian countries. Future research should expand by extensively collecting data from students born and raised in East Asian countries, as well as investigate several other prominent cultural groups within Canada.

Additionally, due to the sensitivity around ethnic identification, we simplified our questions for this study when asking about cultural background. We selectively focused on individuals in Canada who attend the same institution and are proficient in English, to control for these factors. However, future studies may dive deeper and articulate the number of years lived in Canada, immigration status, and related variables. We recognize that this is a limitation and future research should have clear criteria differentiating Canadian-born East Asian individuals and those born in another country. In addition, body image may be a culturally bound construct, therefore the interpretations of survey items may vary across cultures. We must be prudent when collecting data outside of Canada because of these differing cultural norms regarding body image.

Third, specific scenarios considered to be threats to body image should be explored further. The current study asked participants to think of an example from their own personal experience where they faced a threat to their body image however, this may differ significantly from person to person. When investigating cultural differences further, it would be beneficial to compare specific stressful situations involving other people (e.g., at a pool party), vs. body image threats when one is alone (e.g., looking in a mirror) and see if there is any effect on coping strategies used.

Fourth, we discussed the connection between primary and secondary control with Appearance Fixing and Positive Rational Acceptance. The ultimate antecedents for these cultural differences have not been fully investigated within cultural psychology. The cultural differences may be partly due to traditional East Asian beliefs and values rooted in Buddhist philosophies (Keng et al., 2011). Buddhism places an emphasis on acceptance of life challenges and hardships with patience (Deng et al., 2020). The idea of acceptance and surrendering, is viewed as essential in gaining a sense of inner peace to persevere in tough situations. These values of acceptance and desire to regain inner peace may be more prevalent in East Asian Canadians than European Canadians, which could explain their higher usage of Positive Rational Acceptance in response to body image threats.

## 5 Conclusion

East Asian Canadians make up a significantly large portion of the Canadian population yet are highly underrepresented in psychological research. Given the multicultural identity of Canada, it is crucial to avoid merging all the unique cultures existing into one, and instead gain a deeper understanding of their differences. Given their unique values and social orientations, coping mechanisms vary between individuals with East Asian vs. European backgrounds. This study identifies these differences between the selected target groups. The results reveal new findings on cultural differences and offer insight that Appearance Fixing and Avoidance are associated with greater symptoms of stress, depression, and social anxiety. In contrast, Positive Rational Acceptance is negatively associated with these symptoms, resulting in a more positive wellbeing overall. This has implications in the field of cultural psychology, contributing to the research by identifying an additional area in which Eastern and Western cultures differ. Additionally, the current research may be applicable to clinical settings, such as therapy for eating and body image disorders by encouraging practitioners to take a culturally sensitive approach when working with diverse populations. Practitioners would deepen their understanding of the importance of cultural differences not only present between nations, but also within a single, diverse nation like Canada.

## Data Availability

The raw data supporting the conclusions of this article will be made available by the authors, without undue reservation.
