# Supplementary material for: Culture and body image: subcultural variations in coping strategies and their associations with psychological distress among European Canadians and East Asian Canadians
Source: Front Psychol. 2025 Aug 13;16:1596710. doi: 10.3389/fpsyg.2025.1596710 (PMC12382449; doi:10.3389/fpsyg.2025.1596710)
Supplement: Supplementary file 1 [file Supplementary_file_1.pdf]

Supplementary File 1: Multivariate multiple linear regression analysis between coping strategy and symptoms of social anxiety, depression, and stress

A multivariate multiple linear regression was conducted to examine associations between to examine the associations between three coping strategies: Appearance Fixing, Avoidance, and Positive Rational Acceptance (covariates), and six dependent variables (Depression, Stress, Social Interaction Anxiety, Social Phobia, Fear of Negative Evaluation, and Social Avoidance and Distress), while controlling for the fixed effects of Culture and Sex. Collinearity diagnosis indicated that the Variance Inflation Factor (VIF) of Sex, Appearance Fixing, Avoidance, Positive Rational Acceptance to Culture (VIF = 1.10, VIF = 1.16, VIF = 1.10, VIF = 1.01 respectively), Culture, Appearance Fixing, Avoidance and Positive Rational Acceptance to Sex (VIF = 1.04, VIF = 1.10, VIF = 1.08, VIF = 1.03 respectively), Culture, Sex, Avoidance, Positive Rational Acceptance to Appearance Fixing (VIF = 1.03, VIF = 1.03, VIF = 1.03, VIF = 1.02 respectively), Culture, Sex, Appearance Fixing, Positive Rational Acceptance to Avoidance (VIF = 1.05, VIF = 1.09, VIF = 1.13, VIF = 1.03 respectively), Culture, Sex, Appearance Fixing, Avoidance to Positive Rational Acceptance (VIF = 1.03, VIF = 1.10, VIF = 1.18, VIF = 1.09 respectively) were not indicative of multicollinearity.

We chose to use Pillai's Trace as the multivariate test because of its robustness to violations of assumptions, including unequal covariance matrices and deviations from multivariate normality (Olson, 1976). Compared to other multivariate test statistics, Pillai's Trace is typically considered a more robust and conservative option, and therefore is recommended for general use.

Results indicated that the multivariate effect of Appearance Fixing was statistically significant, Pillai's Trace = .140, $F(6, 298) = 8.08$, $p < .001$, $\eta^2_p = .140$. The multivariate effect of Avoidance was also significant, Pillai's Trace = .208, $F(6, 298) = 13.08$ $p < .001$, $\eta^2_p = .208$. The

multivariate effect of Positive Rational Acceptance was also significant, Pillai's Trace = .100, $F(6, 298) = 5.49$, $p < .001$, $\eta^2_p = .100$. The multivariate effect of Culture also showed a significant multivariate effect, Pillai's Trace = .120, $F(6, 300) = 6.770$, $p < .001$, $\eta^2_p = .120$. The multivariate effect of Sex was not statistically significant, Pillai's Trace = .030, $F(6, 298) = 1.55$, $p = .161$, $\eta^2_p = .030$. The interaction between Sex and Culture was statistically significant, Pillai's Trace = .043, $F(6, 298) = 2.23$, $p = .040$, $\eta^2_p = .043$, indicating that European Canadian females are more likely to report higher values for Social Interaction Anxiety (SIAS) compared to males. Such a pattern was not observed among East Asian Canadians.

Results indicated that 25.3% of the variance in Perceived Stress (PSS) scores can be accounted for by the five variables (Culture, Sex, Appearance Fixing, Avoidance, Positive Rational Acceptance) collectively (Adjusted $R^2 = .253$). Looking at the unique contributions of the predictors, the result shows that Appearance Fixing, Avoidance, and Positive Rational Acceptance predicted Perceived Stress (PSS) respectively ($\beta = .136$, $t = 2.87$, $p = .004$), ($\beta = .37$, $t = 6.65$, $p < .001$, ($\beta = -.198$, $t = 3.53$, $p < .001$). The other predictor variables did not significantly contribute towards explaining the association with PSS. Next, 17.5% of the variance in Social Phobia scores can be accounted for by the five variables collectively (Adjusted $R^2 = .175$). Looking at the unique contributions of the predictors, the result shows that Positive Rational Acceptance and Avoidance predicted Social Phobia (SPS), respectively ($\beta = .21$, $t = 2.67$, $p = .008$), ($\beta = .52$, $t = 5.57$, $p < .001$). The other predictor variables did not significantly contribute to explain the association with SPS. Third, 21.5% of the variance in Social Interaction Anxiety (SIAS) scores can be accounted for by the five variables (Culture, Sex, Appearance Fixing, Avoidance, Positive Rational Acceptance) collectively (Adjusted $R^2 = .215$). Looking at the unique contributions of the predictors, the result shows that Appearance Fixing, Avoidance,

and Positive Rational Acceptance predicted Social Interaction Anxiety (SIAS), respectively (β = .15*,* t = 2.34*,* p < .020), (β = .48*,* t = 6.31*,* p < .001), (β = .23*,* t = 2.99*,* p = .003). The other predictor variables did not significantly contribute to explain the association with SIAS. Fourth, 27.3% of the variance in Depression (CES-D) scores can be accounted for by the three variables (Culture, Sex, Appearance Fixing, Avoidance, Positive Rational Acceptance) collectively (Adjusted R^2 = .273). Looking at the unique contributions of the predictors, the result shows that Appearance Fixing, Avoidance, and Positive Rational Acceptance predicted Depression (CES-D) respectively (β = .19*,* t = 2.90*,* p = .004), (β = .54*,* t = 6.85*,* p < .001), (β = -.37*,* t = 4.73*,* p < .001). The other predictor variables did not significantly contribute to explain the association with CES-D. Fifth, 17.2% of the variance in Fear of Negative Evaluation (FNE) scores can be accounted for by the five variables (Culture, Sex, Appearance Fixing, Avoidance, Positive Rational Acceptance) collectively (Adjusted R^2 = .172). Looking at the unique contributions of the predictors, the result shows that Appearance Fixing, Avoidance, and Culture predicted Fear of Negative Evaluation (FNE) respectively (β= -.15*,* t = 3.62*,* p < .001), (β = -.22*,* t = 4.44*,* p < .001), (β = .16*,* t = 2.49*,* p < .013), suggesting that East Asian Canadians are more likely to report higher levels of fear of negative evaluation compared to their European Canadian counterparts. Finally, 19.2% of the variance in Social Avoidance and Distress (SADS) scores can be accounted for by the five variables (Culture, Sex, Appearance Fixing, Avoidance, Positive Rational Acceptance) collectively (Adjusted R^2 = .192). Looking at the unique contributions of the predictors, the result shows that Avoidance, Positive Rational Acceptance, Culture, and Sex predicted SADS (β= .73*,* t = 6.38*,* p < .001), (β = -.456*,* t = 3.95*,* p < .001), (β = -.60*,* t = 3.96*,* p < .001), (β = -.32*,* t = 2.02*,* p = .044), suggesting that East Asian Canadians reported higher

scores of social avoidance and distress compared to European Canadians. This also suggests that females reported higher scores of social avoidance and distress than males.

Overall, this multivariate multiple regression model suggests that Appearance Fixing and Avoidance positively predicts symptoms of social anxiety, depression, and stress. Positive Rational Acceptance negatively predicts most of these symptoms. The contributions of the other predictor variables were less pronounced after accounting for shared variance among all predictors. All of these patterns are consistent with the other models represented in text.

Multivariate Effects

**Effect**	**Pillai's Trace**	**F**	**Hypothesis df**	**Error df**	**p**	**Partial η²**
Appearance Fixing	.140	8.084	6	298	< .001	.140
Avoidance	.208	13.082	6	298	< .001	.208
Positive Rational Acceptance	.100	5.494	6	298	< .001	.100
Culture	.120	6.769	6	298	< .001	.120
Sex	.030	1.550	6	298	.161	.030
Culture × Sex	.043	2.229	6	298	.040	.043

Parameter Estimates

Dependent Variable	Predictor	B	SE	t	p	Partial η²
**SADS**	Appearance Fixing	-.183	.098	1.872	.062	.011
	Avoidance	.733	.115	6.375	<.001	.118
	Positive Rational Acceptance	-.456	.116	3.949	<.001	.049
	Culture	-.601	.152	3.958	<.001	.049
	Sex	-.322	.159	2.022	.044	.013
	Culture x Sex	.218	.223	.977	.329	.003
**FNE**	Appearance Fixing	-.154	.042	3.619	<.001	.041
	Avoidance	-.222	.050	4.441	<.001	.061
	Positive Rational Acceptance	-.021	.050	.414	.679	.001
	Culture	.164	.066	2.488	.013	.020
	Sex	.001	.069	.020	.984	<.001
	Culture x Sex	.130	.097	1.344	.180	.006
**PSS**	Appearance Fixing	.136	.047	2.870	.004	.026
	Avoidance	.371	.056	6.649	<.001	.127
	Positive Rational Acceptance	-.198	.056	3.525	<.001	.039
	Culture	.077	.074	1.042	.298	.004
	Sex	-.081	.077	1.054	.293	.004
	Culture x Sex	-.150	.108	1.388	.166	.006
**SPS**	Appearance Fixing	.212	.079	2.671	.008	.023
	Avoidance	.520	.093	5.571	<.001	.093
	Positive Rational Acceptance	-.131	.094	1.397	.163	.006
	Culture	-.222	.123	1.801	.073	.011
	Sex	-.040	.129	.310	.757	<.001
	Culture x Sex	-.219	.181	1.213	.226	.005
**SIAS**	Appearance Fixing	.149	.064	2.335	.020	.018
	Avoidance	.475	.075	6.312	<.001	.116
	Positive Rational Acceptance	-.226	.076	2.988	.003	.029
	Culture	-.178	.099	1.789	.075	.010
	Sex	-.017	.104	.159	.874	<.001
	Culture x Sex	-.230	.146	1.576	.116	.008

**CESD**	Appearance Fixing	.192	.066	2.904	.004	.027
	Avoidance	.535	.078	6.853	<.001	.134
	Positive Rational Acceptance	-.370	.078	4.728	<.001	.069
	Culture	.098	.103	.949	.343	.003
	Sex	-.132	.108	1.221	.223	.005
	Culture x Sex	-.092	.151	.612	.541	.001

SADS: Social Avoidance and Distress Scale
FNE: Fear of Negative Evaluation
PSS: Perceived Stress Scale
SPS: Social Phobia Scale
SIAS: Social Interaction Anxiety Scale
CESD: Centre Center for Epidemiologic Studies Depression Scale
